# Introduction to the Young African Researchers collection

**DOI:** 10.1039/d4ra90121e

**Published:** 2024-10-21

**Authors:** Abisola O. Egbedina, Jairus L. Lamola, Mina Shawky Adly, Stephen O. Ojwach

**Affiliations:** a Department of Chemistry, University of Ibadan Nigeria; b Faculty of Science, University of Johannesburg, South Africa. Sasol (Pty) Ltd, Research and Innovation 1 Klasie Havenga Rd Sasolburg 1947 South Africa; c Chemistry Department, Faculty of Science, Mansoura University Egypt; d School of Chemistry and Physics, University of KwaZulu-Natal Private Bag X01, Scottsville Pietermariztburg 3209 South Africa Ojwach@ukzn.ac.za

## Abstract

Abisola O. Egbedina, Jairus L. Lamola, Mina Shawky Adly, and Stephen O. Ojwach introduce the *RSC Advances* Young African Researchers collection.
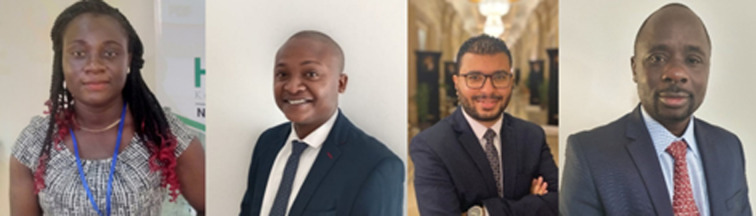

One of the key aims of the Royal Society of Chemistry, and *RSC Advances* in particular, is to promote global scientific research and build capabilities across continents, with a focus on initiatives that support authors from low- and middle-income countries. The majority of manuscripts submitted and articles published in Royal Society of Chemistry journals come from authors based in North America, Europe, China and India. However, this is not to say that there is not enough research output from low- and middle-income countries, and the Sub-Sahara in particular.

*RSC Advances* aims to support researchers at all stages of their career and from a broad range of countries. In 2023, the journal published content from 108 countries. Focusing on contributions from different countries in Africa, just over 600 research articles were published in *RSC Advances* between 2021 and 2023. It is evident that North Africa makes up the largest share of the contributions, with Egypt alone contributing around 55% of the research articles. South Africa provided around 10% of the research articles, with further contributions from Algeria, Botswana, Burundi, Cameroon, Côte d'Ivoire, Ethiopia, Ghana, Kenya, Lesotho, Libya, Malawi, Mali, Morocco, Namibia, Nigeria, Rwanda, Sierra Leone, Sudan, Syria, and Tunisia.

In this collection, we showcase recent publications from African researchers in *RSC Advances*. The articles were selected by our previous Outstanding Student Award winners, Mina Shawky Adly and Jairus Lamola, as well as *RSC Advances* Emerging Investigator Abisola Egbedina. These young African researchers selected their favourite articles representing the high-quality and exciting research we are publishing at *RSC Advances*. The collection was overseen by Associate Editor Stephen Ojwach, who has provided this accompanying Editorial.

In the 20 articles featured in this collection, four papers have joint collaborations with institutions outside the African continent: Germany, Saudi Arabia, India and the USA. In terms of research focus, the articles address and cover a wide spectrum of research interests ranging from materials, biomedical and environmental science, as well as pollution control/water management and catalytic organic transformations (catalysis). The majority of the contributions focus on environmental pollution, specifically water treatment, and materials science for energy production. The work reported is of high quality and industrially relevant. For instance, Ali *et al.* (https://doi.org/10.1039/d3ra02974c) and Shalaby *et al.* (https://doi.org/10.1039/d3ra00168g) report on the design and use of highly sensitive copper nanoparticles and carbon electrodes for the detection of heavy metal cations. The contributions from Abdelmigeed *et al.* (https://doi.org/10.1039/d2ra00936f), Youssef *et al.* (https://doi.org/10.1039/d3ra05729a), Wadie *et al.* (https://doi.org/10.1039/d3ra01211e), and Egbedina *et al.* (https://doi.org/10.1039/D1RA01130H) address the removal of emerging pollutants (pharmaceuticals) from water, and thus demonstrate that despite the lack of resources, Africa is not left behind in cutting edge research, and keeps pace with current technological trends. Hayes *et al.* (https://doi.org/10.1039/d3ra02242k) reports on a state of the art and cost-effective water treatment system using a solar powered hybrid MOF/polymer material. In this area, a review on the recovery of greener biometabolites through sustainable approaches was published by Adeeyo *et al.* (https://doi.org/10.1039/d2ra06596g).

The collection also features research on the design and development of sustainable energy devices. For example, Elisadiki *et al.* (https://doi.org/10.1039/d2ra01322c) and Basuny *et al.* (https://doi.org/10.1039/d3ra06028d) report on the use of plant extract and biomass/biochar composites in the generation of electricity, while Messaoud *et al.* studied Ni/Fe alloys for efficient electrochemical hydrogen production. Msalmi *et al.* and Allah *et al.* studied the use of organically modified Cd and Ni-doped mesoporous carbon hybrid structures as energy storage devices respectively. A more fundamental approach in the syntheses of double layer hydroxides, which are versatile materials in catalysis and in biomedical applications, was reported by Doungmo *et al.* (https://doi.org/10.1039/d2ra05269e) and Hassani *et al.* (https://doi.org/10.1039/d2ra00536k), also utilised starch-coated cellulosic papers as alternative packaging materials in the food industry.

Research in the area of medicinal chemistry and biomedical applications is represented by two articles in this collection. The contribution by Al-Muntaser *et al.* (https://doi.org/10.1039/d3ra00416c) focuses on cancer diagnostics and treatment. The work of Tsaulwayo *et al.* demonstrates the use of metal-based compounds as potential anti-cancer agents and adopts a multidisciplinary approach incorporating inorganic chemistry and biochemistry.

The last research niche covered is in the development of catalysts for fundamental organic transformations, as represented by three articles. For example, Yusuff *et al.* (https://doi.org/10.1039/d1ra09179d), and Lamola *et al.* (https://doi.org/10.1039/d1ra04947j) report the use of sulfonated biochar catalysts in oleic acid esterification and palladium catalysts in Suzuki–Miyaura cross coupling reactions, respectively, while Labyad *et al.* (https://doi.org/10.1039/d3ra05792e) developed a novel eco-friendly and stable freely-available chlorine (FAC) catalyst for the synthesis of terpinic chlorides.

In summary, it is evident that the research contributions and publications in *RSC Advances* from researchers based in Africa have steadily increased over the past few years. The captured articles are of high quality, covering both core and emerging areas such as remediation of emerging pollutants, hydrogen production, and energy and biomedical applications. However, the total numbers are still relatively low compared to those published from North America (around 900), Europe (around 1800), China (around 4500) and India (around 1000). This could be largely attributed to financial implications. However, the Royal Society of Chemistry does offer APC waivers to authors from low- and middle-income countries as part of the Research4Life programme,^1^ so we need to continue to amplify this message to the relevant researchers. More information on our waivers and discounts can be viewed on the *RSC Advances* website.^2^

Promotion of collaborations between African institutions and the west (South-North initiatives) is another route that can increase contributions from the African continent.

We are pleased to present a collection celebrating recent achievements in the chemical sciences in Africa, and we hope this will encourage future researchers to submit to the journal. Please join us in congratulating all our winners for their exceptional achievements. We extend our sincere gratitude to all the authors for their contributions, which has resulted in this wonderful collection.
